# LncRNA SNHG16 contributes to osteosarcoma progression by acting as a ceRNA of miR-1285-3p

**DOI:** 10.1186/s12885-021-07933-2

**Published:** 2021-04-06

**Authors:** Xiao Xiao, Ge Jiang, Shengtao Zhang, Shuo Hu, Yunshan Fan, Gang Li, Haiyang Yu, Shisheng He

**Affiliations:** 1 Department of Orthopedic, Shanghai Tenth People’s Hospital, Tongji University School of Medicine, Shanghai, 200072 China; 2 Spinal Pain Research Institute, Tongji University School of Medicine, Shanghai, 200072 China; 3 Department of Hematology, Shanghai Institute of Hematology, Ruijin Hospital affiliated to School of Medicine, Shanghai Jiaotong University School of Medicine, Shanghai, 200025 China; 4 Department of Orthopedic, The First Affiliated Hospital of Nanchang University, Nanchang, 330006 Jiangxi Province China

**Keywords:** Long non-coding RNA, Small nucleolar RNA host gene 16, Osteosarcoma, Progression, Competing endogenous RNA, microRNA-1285-3p

## Abstract

**Background:**

The long non-coding (lnc) RNA activated by small nucleolar RNA host gene 16 (SNHG16), which has been reported to play a vital role in a number of different types of cancer, is a novel lncRNA. However, following an osteosarcoma (OS) study, the expression pattern, biological roles, clinical values and potential molecular mechanism of SNHG16 remain unclear. In the current study, we aimed to examine its expression and possible function in osteosarcoma (OS).

**Method:**

Cell proliferation was measured by colony formation assay and Cell Counting Kit-8 (CCK-8) in vitro, and xenograft transplantation assay in vivo. Meanwhile, we used transwell chambers to test cell migration and invasion was evaluated. Cell cycle and apoptosis was evaluated by flow cytometry assay. Immunoblotting and qPCR analysis was carried out to detect protein and gene expression, respectively. Luciferase reporter assay was used to predict the potential downstream genes.

**Results:**

The present study demonstrated that SNHG16 is highly expressed in both the tissues of patients with OS, as well as OS cell lines, and its expression level was positively correlated with clinical stage and poor overall survival. Functional assays revealed that the depletion of SNHG16 inhibits OS growth, OS cell progression and promotes apoptosis both in vivo and in vitro. In addition, the present study revealed that microRNA-1285-3p expression levels can be decreased by SNHG16 acting as a ‘sponge’, and that this pathway takes part in OS tumor growth in vivo, and OS cell proliferation, invasion, migration and apoptosis in vitro.

**Conclusions:**

The results from the present study demonstrate the role of lncRNA SNHG16 in OS progression, which is SNHG16 might exert oncogenic role in osteosarcoma (OS) by acting as a ceRNA of miR-1285-3p, and it may become a novel target in OS therapy.

**Supplementary Information:**

The online version contains supplementary material available at 10.1186/s12885-021-07933-2.

## Background

Osteosarcoma (OS) is a type of bone malignancy that most frequently occurs in children and young adults, and is associated with highly aggressive and unfavorable prognoses [1]. Previous studies have indicated that risk factors such as genetic mutations and transcriptional regulatory disorders are key to the development of OS [2]. Treatment including surgical excision, radiotherapy and neoadjuvant chemotherapy has been achieved, but the prognosis of patients with advanced clinical stages at diagnosis remains unfavorable [3, 4]. Therefore, the molecular mechanism underlying OS progression and new molecular biomarkers for early diagnosis require further clarification in order to improve the diagnosis and effective therapy at the onset of the disease.

Long non-coding RNAs (lncRNAs) are a type of RNA of > 200 nucleotides in length, are not protein coding, and play critical roles in the regulation of cell proliferation, apoptosis, migration, invasion, the cell cycle, drug resistance and chromatin remodeling [3–6]. Over the last few years, lncRNAs have gradually been recognized as biological factors in OS. For example, the growth and metastasis of osteosarcoma are promoted by long-non-coding RNA SNHG5 via sponging the miR-212-3p/SGK3 axis [7]. Silencing of lncRNA ANCR suppresses the migration and invasion of osteosarcoma cells by activating the p38MAPK signalling pathway [8]. Furthermore, the resistance of doxorubicin in OS was enhanced by lncRNA FOXC2-AS1 [3]. However, the role of lncRNA in OS pathogenesis has not yet been clearly defined.

Small nucleolar RNA host gene 16 (SNHG16) was originally identified as an oncogene in neuroblastoma, with a poor patient outcome when increased levels of SNHG16 were expressed [9]. Meanwhile, high expression levels of SNHG16 were negatively associated with overall survival time in laryngeal squamous cell carcinoma [10], bladder cancer [11], esophageal squamous cell carcinoma [12] and endometrial carcinoma [13]. Consistent with the outcomes of clinical samples, SNHG16 exhibits a tumorigenic phenotype in cervical [14] and colorectal cancer cell lines in vitro [15]. Finally, knockdown of SNHG16 improved chemosensitivity in bladder cell lines, suggesting that interfering SNHG16 may decrease chemoresistance in patients with bladder cancer [16]. Nevertheless, the potential molecular mechanism and functional role of SNHG16 in OS remains unknown.

miRNAs are endogenous small noncoding RNAs (17–22 nucleotides in length), which take part in tumorigenesis via combination with the 3′ untranslated region (3’UTR) of target genes to interfere with transcription or inhibit translation [17]. Upregulation of miR-1285 could suppress renal carcinoma invasion and migration via regulating TGM2 expression [18], and it can also suppress breast cancer proliferation by regulating TMEM194A expression [19]. The present study confirmed that miR-1285-3p worked as the suppressor gene in OS cell lines and tissues; in constrast, SNHG16 was highly expressed in OS and promoted its progression. Since the theory about competing endogenous RNA (ceRNA) between lncRNA and miRNA is widely accepted [20], the present study demonstrated that SNHG16 contributes to OS progression via acting as the ceRNA of miR-1285-3p.

## Methods

### Clinical samples

A total of 50 patients with OS were recruited into the present study. OS tissues and paired adjacent normal tissues were obtained from the First Affiliated Hospital of Nanchang University (Nanchang, China). All resected specimens were stored at − 80 °C prior to RNA extraction. The present study was reviewed and approved by the Ethics Committee of the First Affiliated Hospital of Nanchang University. All the informed consent was written by patients, and the parent/guardian of patients who were < 18 years old.

### Cell culture

Human OS cell lines (U2OS, MNNG/HOS, 143b, SJSA and MG63), 293 cell line and a normal osteoblast cell line (hFOB 1.19) were obtained from the Cell Bank of the Chinese Academy of Sciences. All cell types were cultured in DMEM (Hyclone; Thermo Fisher Scientific, Inc.) supplemented with 10% fetal bovine serum (FBS) (Gibco; Thermo Fisher Scientific, Inc.) at 37 °C in 5% CO_2_ and 95% air.

### Plasmid construction

The SNHG16 fragment containing the miR-1285-3p binding site was amplified and cloned into the *Not*l and X*ho*I restriction sites of the psiCHECK2 Vector (Promega Corp) in order to obtain the reporter vector psiCHECK2-SNHG16-wild-type (SNHG16-wt). Mutating the miR-1285-3p binding sites in SNHG16 resulted in the psiCHECK2-SNHG16-mutant-type vector (psiCHECK2-SNHG16-mut).

### Cell transfection

Two different small interfering (si)RNAs against SNHG16 were designed and synthesized by GenePharma, and transfected into U2OS and MNNG/HOS cells using Lipofectamine® 3000 (Invitrogen; Thermo Fisher Scientific, Inc.) according to the manufacturer’s protocol. Cells were collected 48 h after transfection, and the knockout efficiency was detected via reverse transcription-quantitative PCR (RT-qPCR). The sequences of SNHG16 siRNA were as follows: SNHG16 siRNA, forward, 5′-CCUGGGUAUAAUCUCACAATT-3′, and reverse, 5′-UUGUGAGAUUAUACCCAGGTT-3′; SNHG16 siRNA, forward, 5′-GGAACAUACUGCUAUCAUATT-3′, and reverse, 5′-UAUGAUAGCAGUAUGUUCCTT-3′; miR-1285-3p mimic, forward, 5′-UCUGGGCAACAAAGUGAGACCU-3′, and reverse, 5′-GUCUCACUUUGUUGCCCAGAUU-3′; miR-1285-3p inhibitor, forward, 5′-AGGUCUCACUUUGUUGCCCAGA-3′.

### RT-qPCR

Total RNA was extracted from OS tissues of cells using TRIzol® reagent. RT was performed using PrimeScript™ RT reagent kit (Takara Bio). The RT conditions were 15 min at 37 °C and 5 s at 85 °C, and the mixture was subsequently stored at 4 °C. The qPCR primers used were as follows: U6 forward, 5′- TTACATTGCTATCCACAGAACGG − 3′, and reverse, 5′-CTATGCTGCTGCTTTTTGCTC-3′; miR-1285-3p forward, 5′-GCGTCTGGGCAACAAAGTG-3′, and reverse, 5′-AGTGCAGGGTCCGAGGTATT-3′; 18S forward, 5′-GAAACGGCTACCACATCC − 3′, and reverse, 5′-ACCTCCCGTTCAGACCA-3′; SNHG16 forward, 5′-TACTCTGTTGGAAGAGCCTAA-3′, and reverse, 5′-GGGTGTTGGTAACGAAA − 3′. By the way, the short length of miRNA is not suitable for the use of bidirectional primers, we use RT-primer to prolong their sequence and quantify them. The reverse transcription primer sequence is composed of a 5′-end stem-loop structure and a 3′-end specific sequence. The primer sequence is about 42–44 nucleotides; the 36 nucleotide sequence at its 5 ‘end is fixed as stem-loop, and the 6–8 nucleotide structure at its 3’ end are complementary to microRNA. Firstly, we can get the mature sequence of microRNA through miRbase (http://www.mirbase.org). Then, we download the sequence and put them into the “miRNA RT primer”, a primer design software, to get the reverse primer sequence. Finally, the reverse and the forward primer are producted by primer3plus, an online primer design website (http://www.primer3plus.com).

### Western blotting

OS tissues and cells were harvested and then split using RIPA protein extraction reagent (Beyotime Institute of Biotechnology). Proteins were transferred onto PVDF membranes, and then incubated with the specific primary and secondary antibodies. Autoradiogram and chemiluminescence were used to quantify the densitometry, and GAPDH was used as the control. Antibodies GAPDH, pro-caspase3, cleaved-caspase3, bcl-2 and bax were purchased from Abcam.

### Cell proliferation assays

The change in cell viability following transfection was assessed using a Cell Counting Kit-8 (CCK-8; Dojindo Molecular Technologies) assay. In brief, OS cell lines were equally seeded into 96-well plates following treatment with different types of siRNA, and the cells were cultured with CCK8 reagent for 2 h. The optical density (OD) was measured every 24 h using at 450 nm, according to the manufacturer’s protocol.

### Cell migration and invasion assays

In the cell migration and invasion assays, Transwell chambers were used (Costar; Corning, Inc.). For the migration assay, 5 × 10^4^ cells were added to the upper chambers with 200 μl FBS-free medium, while 500 μl medium supplemented with 10% FBS was injected into the lower chambers. For the invasion assay, the upper chamber was pre-coated with Matrigel (BD Biosciences), and 1 × 10^5^ cells were plated in the upper chamber cultivated in 200 μl FBS-free medium. The lower chamber was filled with complete medium. After 24 h incubation, the traversed cells were stained with crystal violet. All images are observed under the microscope (Leika) and taken by Leica LAS AF Lite software. The image resolution is at least 300ppi of each image.

### Colony formation assay

Cells (1 × 10^3^/well) were seeded in 12-well plates and incubated in complete medium. The colonies were fixed with 10% formaldehyde for 10 min and stained with 1% crystal violet after 2 weeks. The colony number was counted manually.

### Cell cycle and apoptosis analysis

In the cell cycle analysis, propidium iodide (PI) was used to stain OS cells, and a Cycle Reagent kit (BD Biosciences) was used for the analysis. In the apoptotic analysis, cells were stained with PI and Annexin V-FITC. Flow cytometric analysis was performed with a flow cytometer (BD Biosciences) 48 h after cell transfection.

### Luciferase reporter assay

First, psiCHECK2-SNHG16-wt or the mutant type with miR-1285-3p mimics were co-transfected into MNNG and cells using Lipofectamine® 3000 (Invitrogen; Thermo Fisher Scientific, Inc.). After 48 h, Renilla luciferase activity was assessed and used as a measure of normalization to compare the relative luciferase activity.

### Xenograft transplantation

Ten male nude (BALB/c) mice (4 weeks old, average 15 g), (which bought from Xipuer-Bikai Laboratory Animal Technology Company) were randomly divided into two groups method, and there are five mice in each group. The condition of the experiment in temperature is 22 ± 2 °C, and the humidity is 45–55%. 12 h of light is provided per day, and each mice can eat and drink freely. By the way, we pay highly attention to the health status and activity of the mice in the cage during the study, and the room is always well ventilated, and the environment is quiet. For SNHG16, first, we transduced MNNG/HOS cells with a lentiviral vector sh-SNHG16 or sh-NC, which constitutively expressing green florescent protein (GFP) gene. Following the transduction, MNNG/HOS luciferase cells were select by the puromycin, and maintained in culture. MNNG/HOS cells stably expressing sh-SNHG16 or negative control were amplified and 1 × 107 cells were subcutaneously injected into the right flank of male nude (BALB/c) mice. Tumor growth and tumor volumes was measured twice a week. After 6 weeks, all mice were sacrificed by cervical spinal dislocation and tumors were excised. The animal studies were approved by the Ethics Committee of Shanghai Tenth People’s Hospital.

### Statistical analysis

All statistical analyses were performed using SPSS 20.0 software (IBM). Data are expressed as the Mean ± SD for at least three separate experiments. Differences between groups were analyzed using the Student’s t test or one-way ANOVA. The Kaplan-Meier method and a Cox proportional hazard regression model were used for the prognostic analysis, *p* values < 0.05 were considered statistically significant.

## Results

### SNHG16 is upregulated in OS tissues and cells, and is associated with poor prognosis

**Fig. 1:**
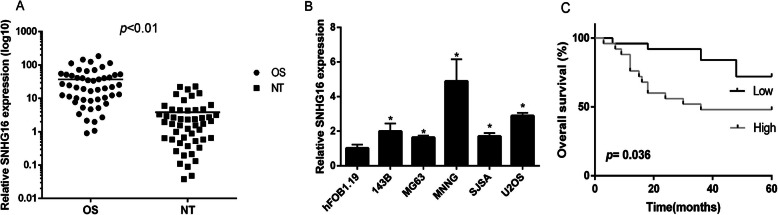
SNHG16 expression in OS tissues and cell lines. (A). The SNHG16 expression in 50 pairs of OS and noncancerous tissues(NT). (B). The SNHG16 expression in five OS cell lines (U2OS, MNNG, SJSA, MG-63 and 143b) and normal osteoblast cells (hFOB1.19). (C). Kaplan-Meier analyses of the association between SNHG16 expression and overall survival(*p < 0.05)

The present study performed RT-qPCR in order to determine the differential expression levels of SNHG16 in OS tissues and paired adjacent non-tumor bone tissues from 50 patients. Furthermore, the expression levels of SNHG16 were also determined in MNNG/HOS, U2OS, MG-63, SJSA, 143b and hFOB 1.19 cells. SNHG16 expression was increased in OS tissues and cell lines compared with adjacent non-tumor bone tissues and the normal OS cell line (Fig. 1a and b). Kaplan-Meier analysis demonstrated that high expression levels of SNHG16 were significantly associated with poor prognosis (Fig. 1c). The association between clinical significance and SNHG16 expression levels in patients with OS was also analyzed. There were no significant differences between SNHG16 expression and age, sex, tumor size or tumor location (Table 1). However, it was revealed that SNHG16 expression in OS was associated with clinical stage (*P* = 0.037).

**Table 1:** Correlation between SNHG16 expression and clinicopathological features in OS patients

Variables	SNHG16 expression levels		*P*
	Low	High	
Sex			
Male	17	12	0.152
Female	8	13	
Age			
≤ 20	10	13	0.395
> 20	15	12	
Location			
Femur/Tibia	21	19	0.480
Elsewhere	4	6	
Tumor size (cm)			
≤ 5	8	12	0.248
> 5	17	13	
Clinical stage			
I + IIA	12	5	0.037
IIB/III	13	20	
Distant Metastasis			
Yes	7	9	0.544
No	18	16	

*P* value was acquired by Pearson chi-square test. The median expression level was used as the cutoff

### Knockdown of SNHG16 inhibits proliferation, migration and invasion in OS cells

**Fig. 2:**
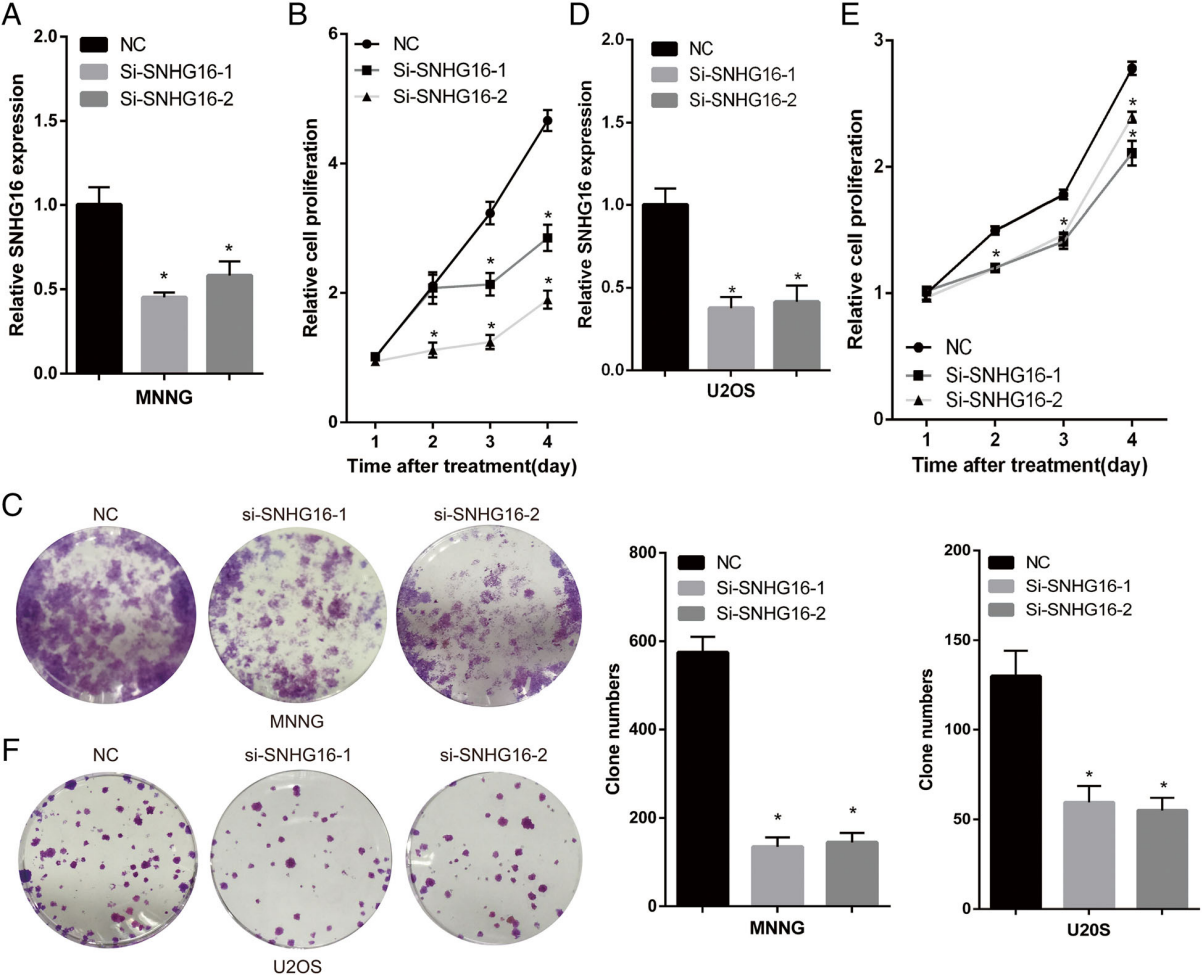
SNHG16 enhanced OS cells proliferation in vitro, (A) (D) The relative expression of SNHG16 in negative control and SNHG16 silenced MNNG (or U2OS) cells. (B) (E) The cell growth rates were determined by performing CCK-8 assay. Knockdown SNHG16 in MNNG (or U2OS) cells suppressed cell proliferation. (C) (F) Colony formation assay of control and SNHG16 silenced MNNG (or U2OS) cells. Representative graphs are shown. Results are shown as mean ± SD;*P<0.05 (ANOVA)

**Fig. 3:**
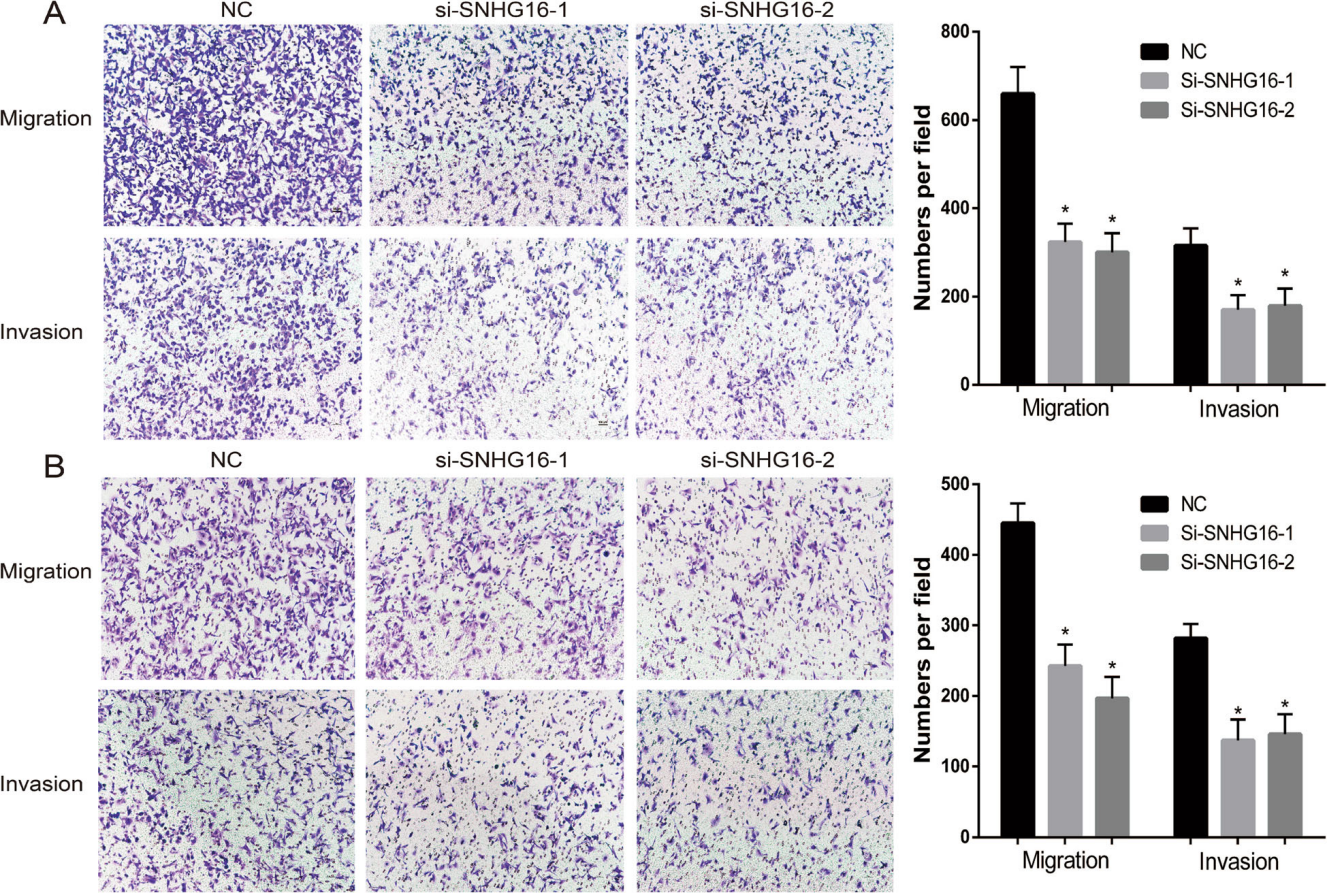
SNHG16 enhanced OS cells migration and invasion in vitro and tumor growth in vivo. MNNG (A) U2OS(B). The images and number of the migrated and invaded MNNG (and U2OS) cells

In order to investigate the functional role of SNHG16 in the progression of OS, SNHG16 siRNA and negative control were transfected into MNNG/HOS and U2OS cells. The expression level of SNHG16 was tested using RT-qPCR 48 h post-transfection, and the results were compared with non-specific siRNA transfection. SNHG16 siRNA transfection resulted in significant knockdown of SNHG16 expression (Fig. 2a and d). The effect of SNHG16 on OS proliferation was determined using a CCK-8 assay. In MNNG/HOS and U2OS cells, SNHG16 knockdown led to decreased cell proliferation compared with that in non-transfected control (NC) cells (Fig. 2b, c, e and f). Transwell assays were then used to assess the effect of SNHG16 on OS migration and invasion. As presented in Fig. 3a and b, the results revealed the positive effect of SNHG16 knockdown on MNNG/HOS and U2OS cell migration and invasion.

### SNHG16 blocks the cell cycle and accelerates apoptosis in OS cells

**Fig. 4:**
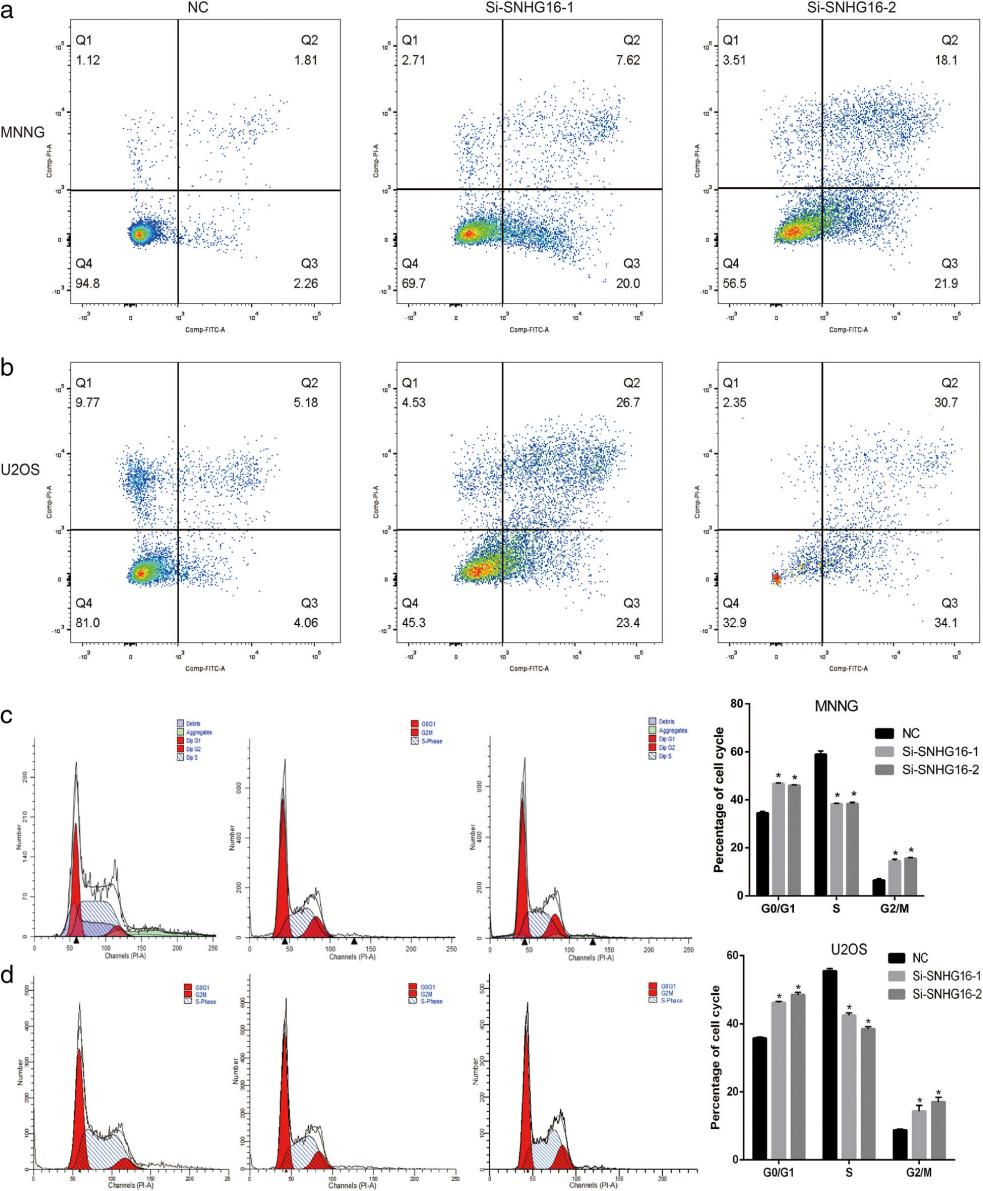
SNHG16 blocked the cell cycle and accelerated apoptosis in osteosarcoma cells. (**a**) (**b**) Compared with negative controls, knockdown SNHG16 resulted in increased apoptosis and decreased surviving proportion of MNNG or U2OS cells. (**c**) (**d**) SNHG16 knockdown in the MNNG or U2OS cells significantly increased the rates of G2/M phase relative to the Si-NC group. * *P* < 0.05

Flow cytometry analyses were used to measure the effect of SNHG16 and miR-1285-3p on the cell cycle and apoptosis. SNHG16 knockdown in the MNNG/HOS and U2OS cells significantly increased the number of cells in the G_2_/M phase compared with the negative controls (Fig. 4C and D). Furthermore, compared with the si-NC group, knockdown of SNHG16 resulted in an increase in the proportion of apoptotic cells in both U2OS and MNNG/HOS cells, compared with the negative controls (Fig. 4A and B).

### SNHG16 knockdown inhibits OS growth in vivo

**Fig. 5:**
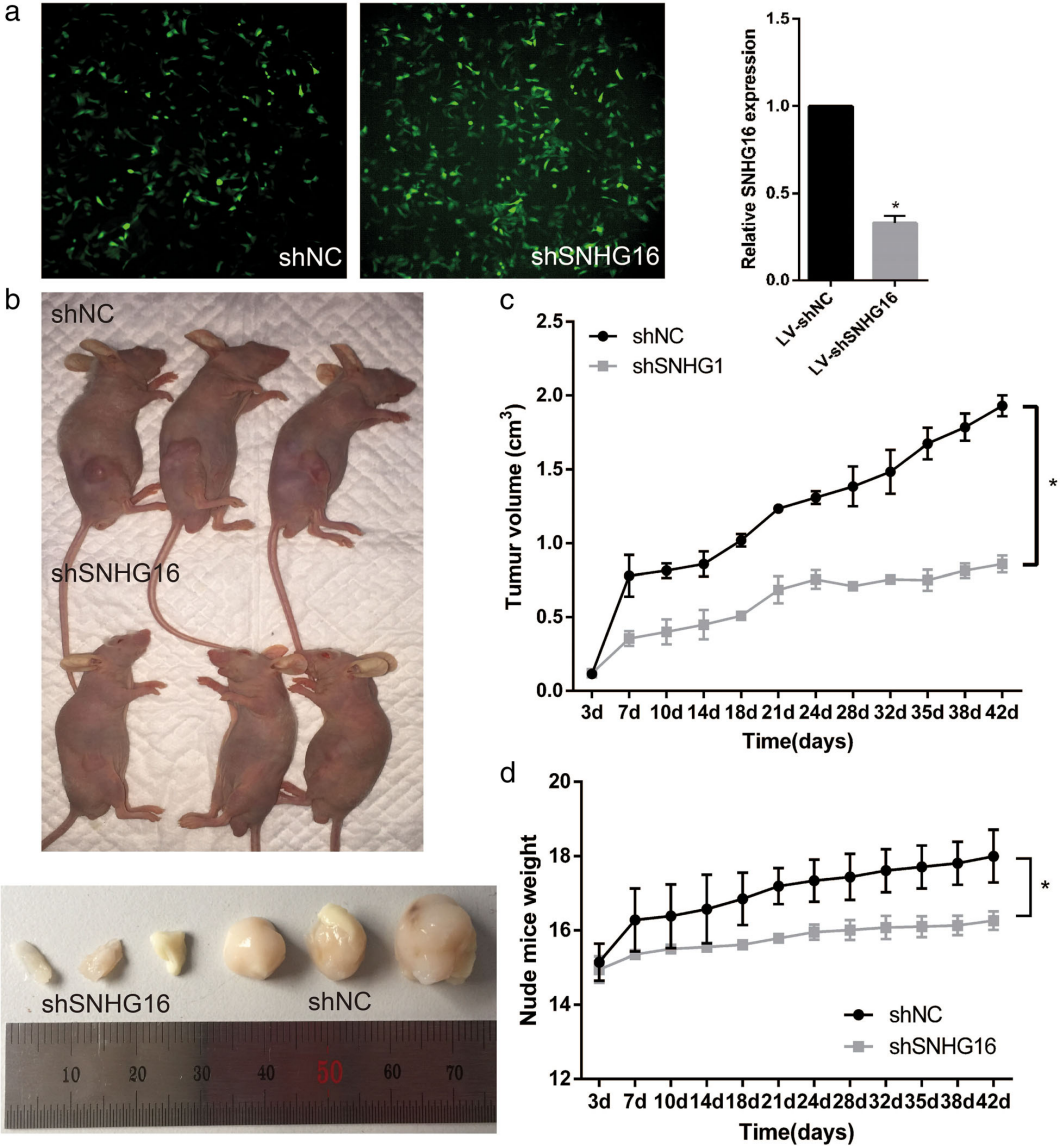
(**a**). Immunofluorescence detection of GFP MNNG cells transduced with lentivirus (LV) expressing GFP showed high transduction efficiency. (**b**). Expression of SNHG16 is significantly blocked compared with the shNC. (**c**). The nude mice were killed and tumor were measured. (**d**) (**e**). Volumes and weights of transplanted tumors, and in sh-SNHG16 group, tumor growth were restricted. * *P* < 0.05

In order to demonstrate the role of SNHG16 on tumor growth in vivo, MNNG cells were stably transduced with lentivirus SNHG16-shRNA (sequence same as si-SNHG16–2) and lentivirus shNC. The high GFP expression in transduced cells showed in Fig. 5A, and the transduction efficiency is presented in Fig. 5B. The mean weights and volumes of xenograft tumors generated from sh-SNHG16 MNNG cells were lower than those of shNC cells (Fig. 5C-E).

### SNHG16 may be a target of miR-1285-3p

**Fig. 6:**
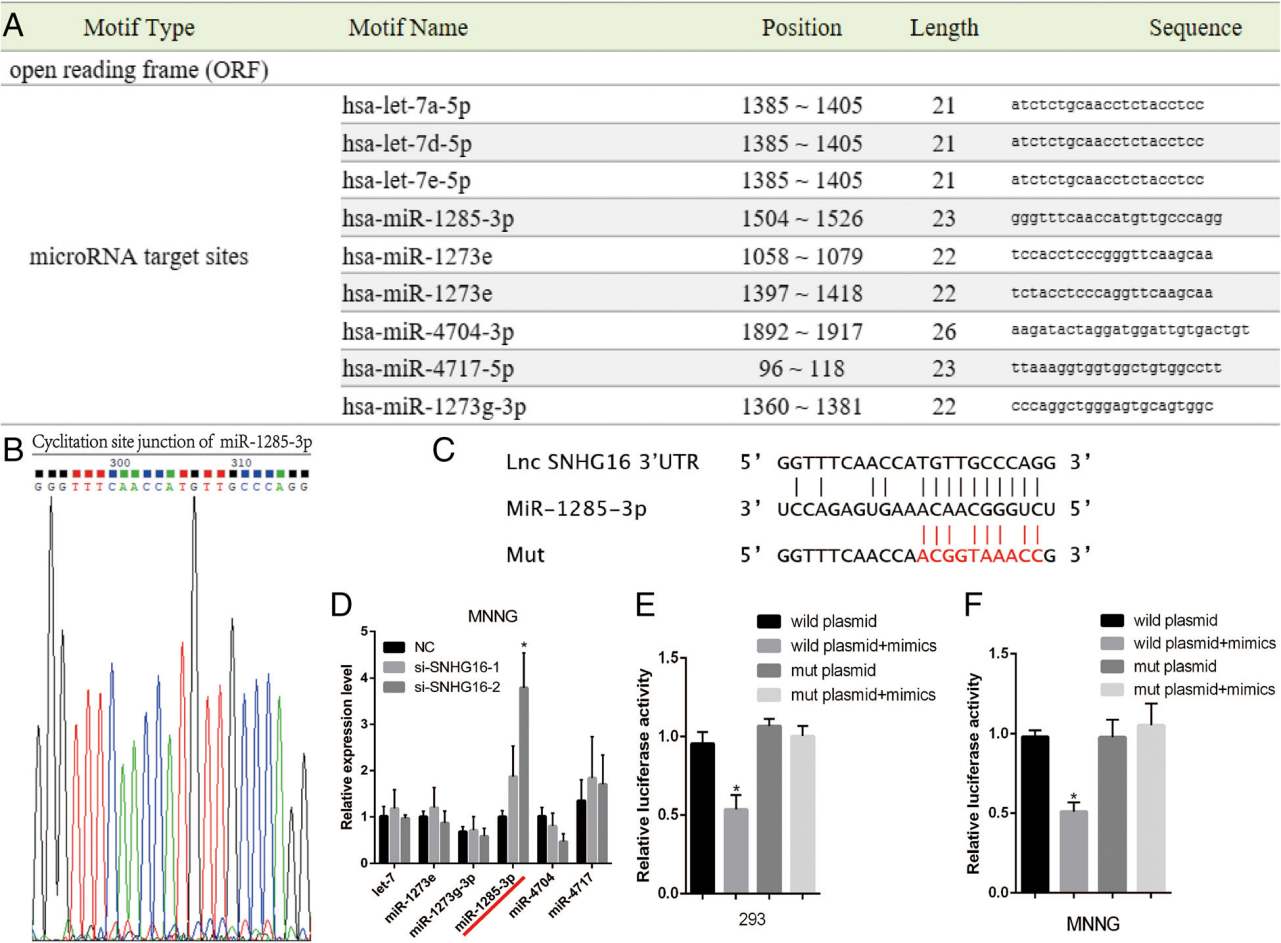
SNHG16 directly interacted with miR-1285-3p in OS cells. (A) The results of miRNAs related to SNHG16 by bioinformatics analysis. (B) The qPCR products were used to confirm the binding site of SNHG16 sequence. (C) The sequence between miR-1285-3p response element (MRE) and SNHG16. (D) Among these miRNAs, only miR-1285-3p was negatively associated with the expression of SNHG16. (E) (F) The luciferase activity of SNHG16-wt was repressed by overexpression of miR-1285-3p, while the mutant type was not affected in 293 and MNNG cells. * *P* < 0.05

**Fig. 7:**
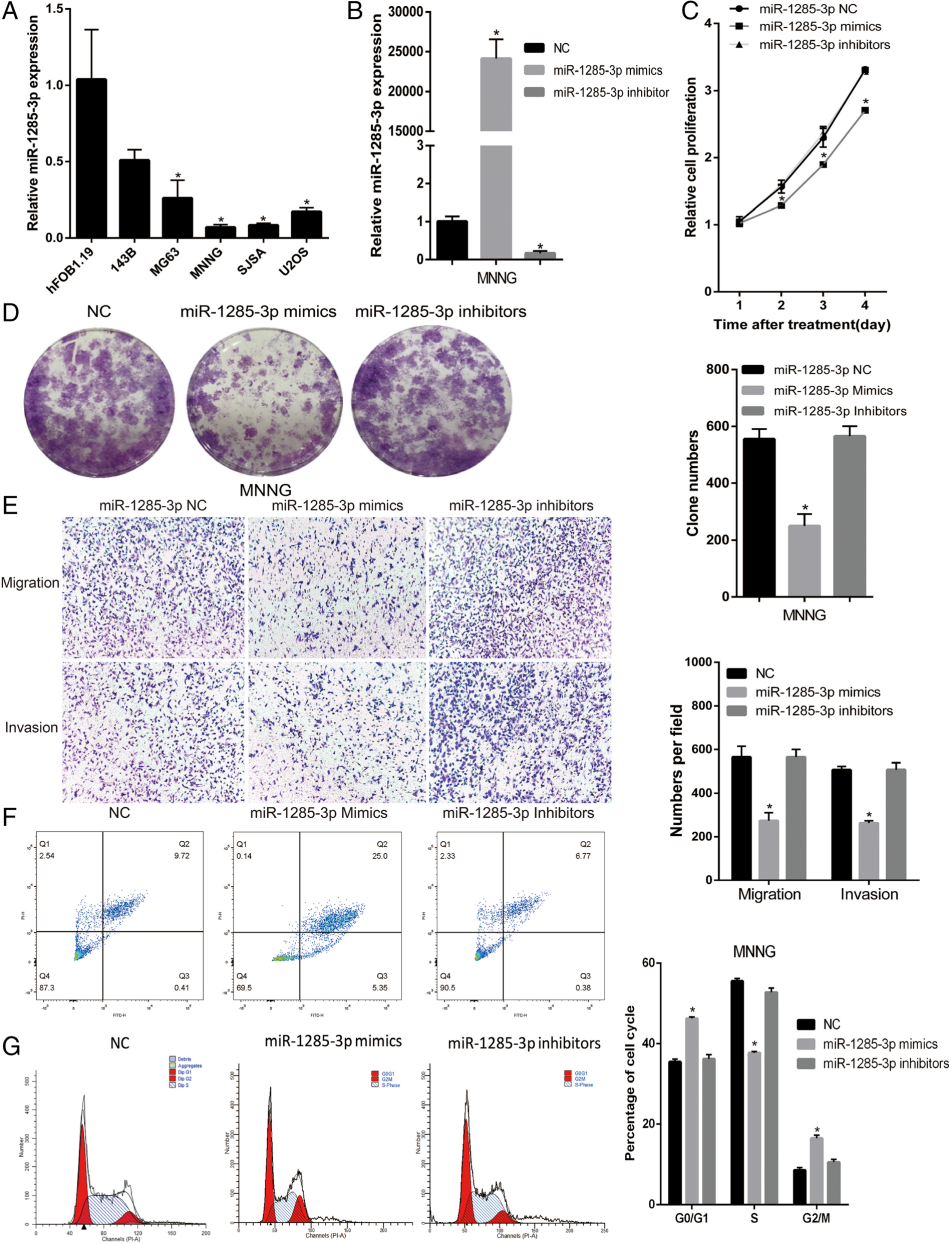
SNHG16 was directly targeted by miR-1285-3p. (A) The miR-1285-3p expression in five OS cell lines and a normal osteoblast cells. (B)The relative expression of miR-1285-3p mimics, inhibitors and negative control were detected in MNNG cells. (C) (D) Cell proliferation is suppressed by miR-1285-3p mimics. (E) Upregulated miR-1285-3p reduced migration and invasion of MNNG cells. (F) (G) Increasing miR-1285-3p in the MNNG cells promoted the rates of G2/M phase relative to the NC group, and enhancing expression of miR-1285-3p resulted in increased apoptosis

Various published articles have demonstrated that lncRNAs may act as ceRNAs through microRNA response element s(MRE) binding to miRNA. In the present study, it was investigated whether SNHG16 affected OS progression through ceRNAs and the miR-1285-3p pathway. First, bioinformatics analysis (RegRNA 2.0) was performed in order to investigate the potential combing site of SNHG16 (Fig. 6a and b), and miR-1285-3p was selected as a candidate (Fig. 6c and d). Secondly, the RT-qPCR analysis verified that miR-1285-3p levels were lower in OS cell lines, and could participate in OS regulation (Fig. 7). Thirdly, a luciferase reporter assay was used to assess whether miR-1285-3p could target SNHG16. According to the binding sites in SNHG16, plasmids were constructed that contained wild-type and mutant miR-1285-3p (psiCHECK2-SNHG16-wt and psiCHECK2-SNHG16-mut, respectively). The results revealed that miR-1285-3p mimics could significantly weaken the fluorescence signal of psiCHECK2-SNHG16-wt in 293cells (Fig. 6e), but no effect was observed if the binding sites in SNHG16 were mutated (co-transfection of miR-1285-3p mimics and psiCHECK2-SNHG16-mut). In MNNG/HOS cells the phenomenon was the same (Fig. 6f). The expression level of SNHG16 and miR-1285-3p were then regulated in order to analyze their influence on each other. When silencing SNHG16, the expression level of miR-1285-3p was increased (Fig. 6d). Meanwhile, in the group of miR-1285-3p mimics, the rate of cells remaining in the G_2_/M phase was elevated and the apoptosis proportion was increased when compared with the group of miR-1285-3p inhibitors. That is to say, the phenomenon of cell cycle and apoptosis was also negatively altered between SNHG16 and miR-1285-3p.

### SNHG16 exerts its function in OS cells in a similar manner to miR-1285-3p

**Fig. 8:**
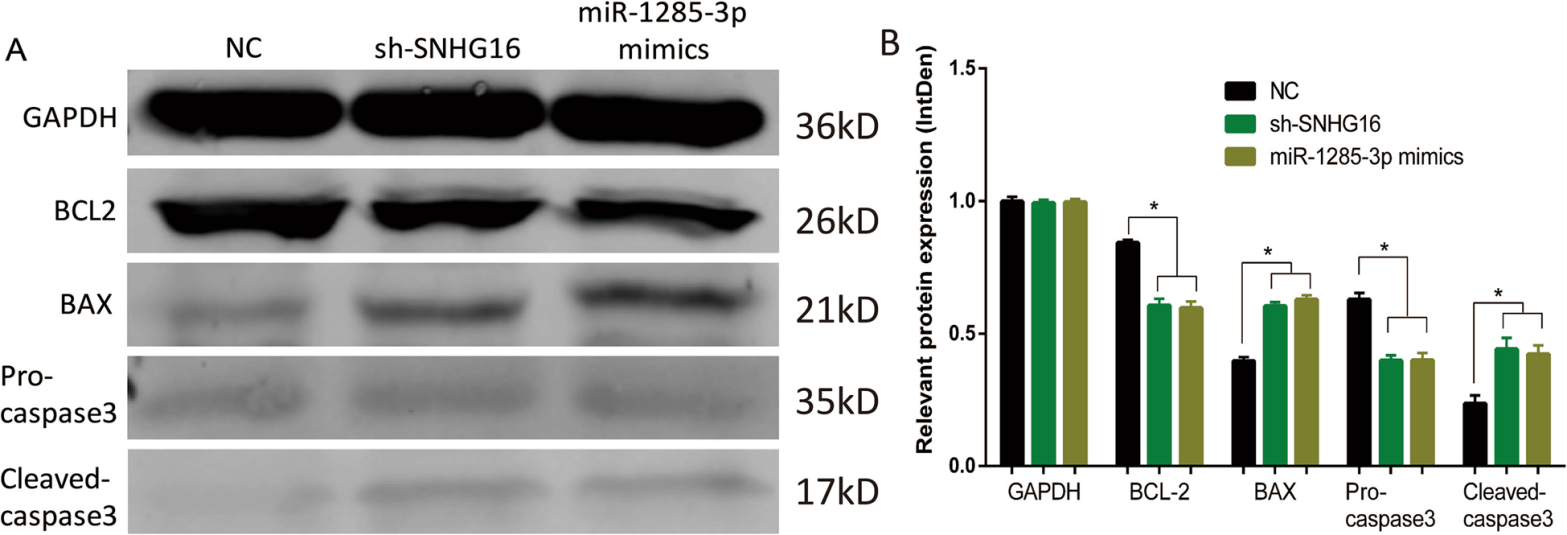
The function of shSNHG16 was reversed by miR-1285-3p inhibitor both in (A) CCK-8 assay and (B) Transwell assay. * *P* < 0.05

In order to determine the functional role of the interaction between SNHG16 and miR-1285-3p in OS, the present study investigated the expression level of miR-1285-3p in OS cell lines (Fig. 7a), and tested the influence of miR-1285-3p in MNNG (Fig. 7b-g). Rescue experiments were then applied in order to identify the influence of SNHG16 in OS proliferation and migration through targeting miR-1285-3p. miR-NC or miR-1285-3p inhibitor was transfected into shNC or shSNHG16 cells, and the effect of SNHG16 in OS growth and migration could be partly reversed by miR-1285-3p inhibitor (Fig. 8a and b). These data illustrated that SNHG16 enhanced cell proliferation and migration via targeting miR-1285-3p.

### SNHG16 silencing increases the protein expression levels of cleaved-caspase-3 and Bax, and decreases Bcl-2 and pro-caspase-3 protein expression

**Fig. 9:**
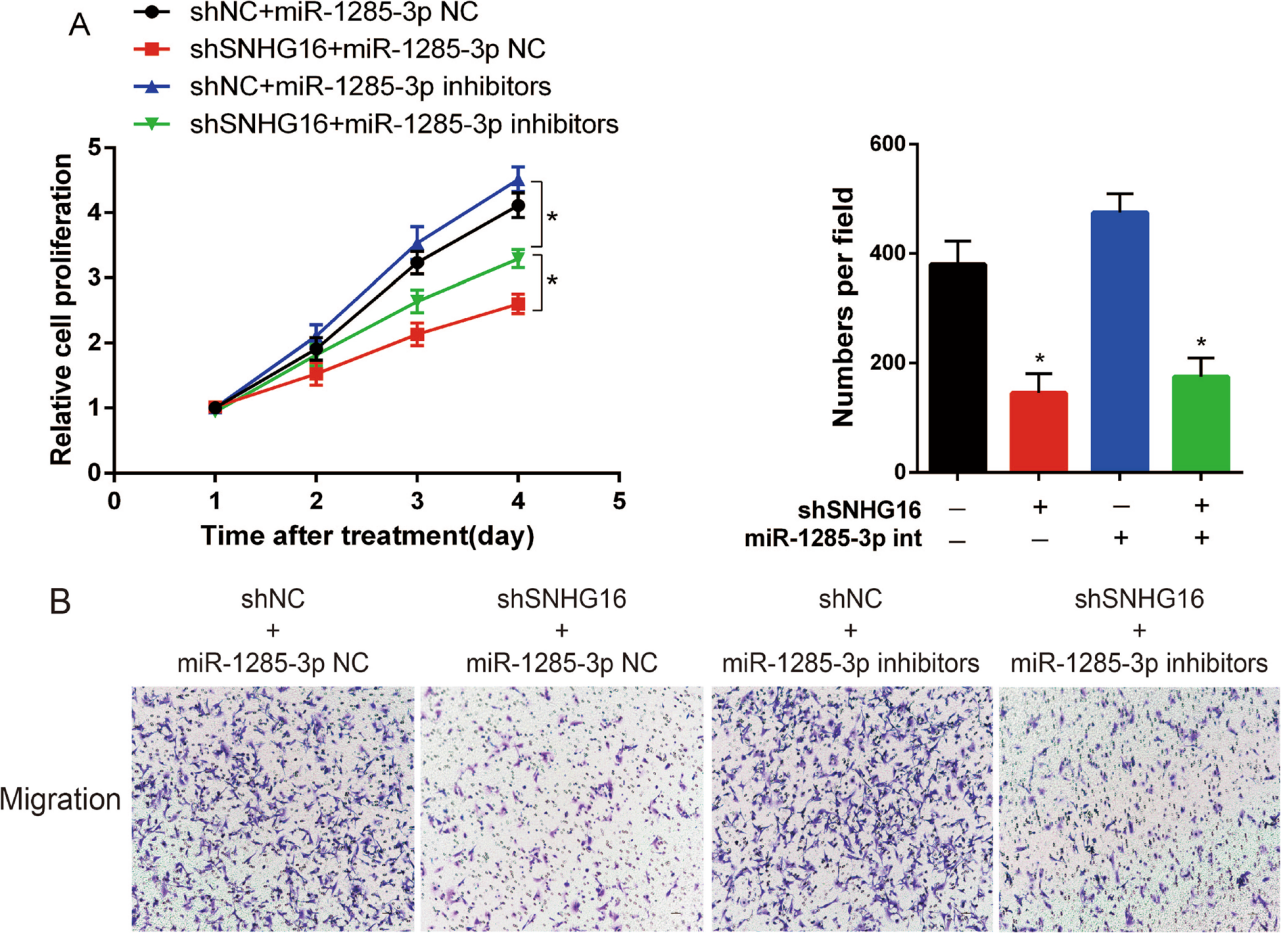
(A) The protein level was shown above. (B) The density of each group. * *P* < 0.05. Full-length blots/gels are presented in Supplementary Figure [wb origin]

Western blotting analysis was applied to measure the protein levels of cleaved-caspase-3, pro-caspase-3, Bax and Bcl-2 among the following groups. As demonstrated by the results of this analysis (Fig. 9), compared with the negative control, the sh-SNHG16 and miR-1285-3p mimics groups exhibited increased protein levels of Bax and cleaved-caspase-3, and decreased expression of pro-caspase-3 and Bcl-2. These findings revealed that silencing of SNHG16 increases the protein levels of cleaved-caspase-3 and Bax, and decreases pro-caspase-3 and Bcl-2 protein expression, supporting the notion that SNHG16 inhibits apoptosis by sponging miR-1285-3p in OS cells.

## Discussion

Increasing evidence has confirmed that various lncRNAs are dysregulated in a number of different types of cancer, including gastric [21], lung [22], breast [23], and colorectal cancer [24]. Recent research has demonstrated that lncRNAs participate in the development of OS by regulating cell proliferation, migration, metastasis and apoptosis. For example, Perry et al [25] highlighted that the PI3K/mTOR pathway plays a fundamental part in maintaining cell viability in OS. Wang et al [26] revealed that lncRNA AK093407 promotes STAT3-mediated proliferation and inhibits OS apoptosis Zhang et al [27] demonstrated that lncRNA ODRUL regulates the progression of OS via miR-3182/MMP2 Axis Wang et al [28] reported that lncRNA DANCR promotes OS proliferation and metastasis via ROCK1 activation, through regulating the expression of miR-335-5p and miR-1972. SNHG16 has been reported to play a functional role in the progression of numerous different types of cancer, such as gastric cancer [29] and cervical cancer [30].

In the present study, the expression levels of a novel lncRNA SNHG16 were analyzed in OS tissues and their paired adjacent non-cancerous tissues. It was revealed that SNHG16 was increased in both OS tissues and cell lines, and was closely associated with clinical stage and poor outcome in OS. The present study indicated the functional role of SNHG16 in OS cells, in that SNHG16 knockdown could suppress cell proliferation, migration, invasion and promoted apoptosis in OS cells in vitro, as well as inhibit tumor growth in vivo; however, the regulation of SNHG16 expression remains unclear. As aforementioned, the present study first demonstrated the functional significance of SNHG16 expression in OS, and the results suggested that SNHG16 functions as an oncogene and promotes OS malignant progression. Therefore, SNHG16 may be a promising prognostic and diagnostic marker and therapeutic target for OS.

It has been suggested that lncRNAs could indirectly harbor miRNAs to decrease expression and activity by acting as a ‘sponge’. Therefore, the present study performed a bioinformatics analysis and demonstrated the binding sites between SNHG16 and miR-1285-3p via a luciferase assay. The results suggested that SNHG16 may exert functions by sponging miR-1285-3p. Nevertheless, the function of miR-1285-3p in OS remains uncertain. Previous studies have demonstrated that miR-1285-3p participates in the tumorigenesis of pancreatic cancer [31] and ovarian cancer [32]. The results of the present study indicated that miR-1285-3p was significantly decreased in OS cells. Furthermore, it was demonstrated that miR-1285-3p could promote apoptosis and inhibit OS cell proliferation, migration and invasion in vitro. The present study implies that miR-1285-3p may play a functional role in different types of cancer.

Overall, the present study first demonstrated that, in OS tissues and cell lines, the expression level of lncRNA SNHG16 is upregulated. High expression levels of SNHG16 in OS are closely associated with poor clinical outcome and advanced disease stage. Furthermore, it was also indicated that SNHG16 promotes OS tumor growth in vivo, OS cell invasion, migration and proliferation, and suppresses apoptosis in vitro via association with miR-1285-3p. These results validated that the interaction between SNHG16 and miR-1285-3p could be a novel pathway involved in various different types of cancer, and that this mechanism could provide a promising therapeutic target, particularly in the treatment of OS. By the way, miRNA generally acts on mRNA to regulate the transcription of target genes, and it is a limitation that the we did not study the target gene protein molecules regulated by miR-1285-3p, and we plan to test it in further investigations.

## Conclusions

In summary, our results from the present study demonstrate that the expression level of lncRNA SNHG16 is increased in OS cell lines compared to that in normal osteoblast cell lines. Function experiment further showed that lncRNA SNHG16 may promote the proliferation, migration, invasion and reduce apoptosis by inhibiting the expression of miR-1285-3p, which may become a novel target in OS therapy.

## Supplementary Information

**Additional file 1.** WBR

**Additional file 2.** Multivariate analysis

**Additional file 3.** GAPDH

**Additional file 4.** BCL2

**Additional file 5.** BAX

**Additional file 6.** Pro-caspase

**Additional file 7.** Cleaved-caspase

## Data Availability

The datasets used or analyzed during the present study are available from the first author or corresponding author upon reasonable request.

## Abbreviations

LncRNAs: Long non-coding RNAs
SNHG16: Small nucleolar RNA host gene 16
OS: osteosarcoma
CCK-8: Cell Counting Kit-8
ceRNA: Competing endogenous RNA
RT-Qpcr: Reverse transcription-quantitative Polymerase Chain Reaction
OD: Optical density
GFP: Green florescent protein
GAPDH: Glyceraldehyde-3-phosphate dehydrogenase
Bcl2: B-cell lymphoma-2
BAX: Bcl2-Associated X
