# Supplementary material for: LncRNA SNHG16 contributes to osteosarcoma progression by acting as a ceRNA of miR-1285-3p
Source: BMC Cancer. 2021 Apr 6;21:355. doi: 10.1186/s12885-021-07933-2 (PMC8022398; doi:10.1186/s12885-021-07933-2)
Supplement: Supplementary file 1 — Additional file 1. WBR [file 12885_2021_7933_MOESM1_ESM.docx]

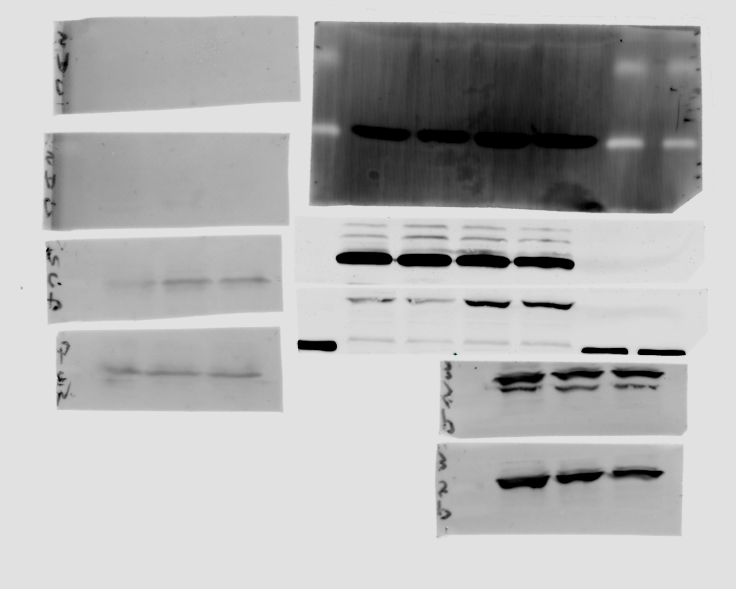

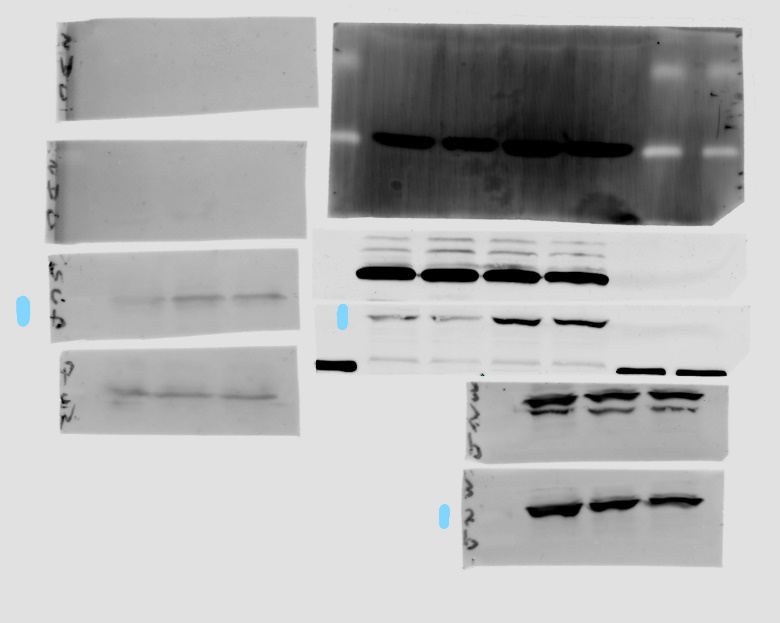

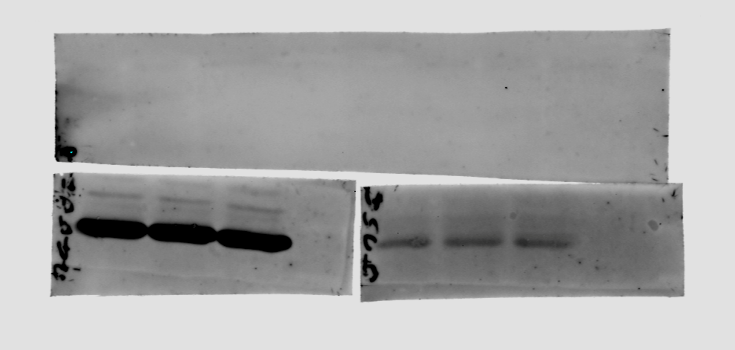

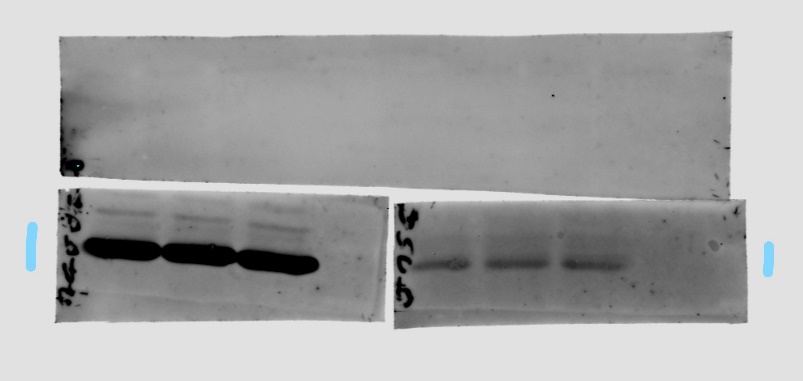


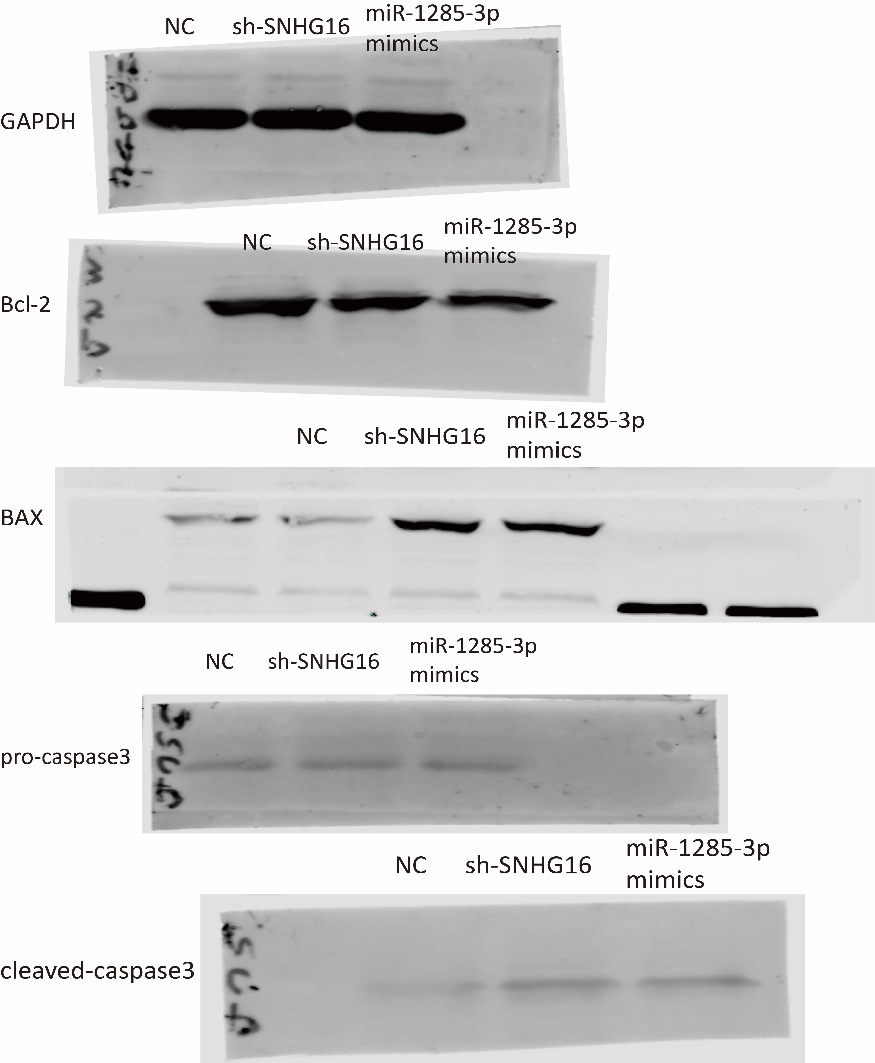


**Figure legends**

All the original, full-length gel and blot images was shown above, and all the samples were labelled as the last figure. Figure were taken by odyssey software
