# Supplementary material for: LncRNA SNHG16 contributes to osteosarcoma progression by acting as a ceRNA of miR-1285-3p
Source: BMC Cancer. 2021 Apr 6;21:355. doi: 10.1186/s12885-021-07933-2 (PMC8022398; doi:10.1186/s12885-021-07933-2)
Supplement: Supplementary file 2 — Additional file 2. Multivariate analysis [file 12885_2021_7933_MOESM2_ESM.docx]

Supplementary Table1. Univariate analysis of variables in patients with osteosarcoma using Kaplan-Meier method.

| Category | Overall Survival  (log-rank *p* value) |
| --- | --- |
| SNHG16 expression | 0.036 |
|  |  |
| Sex | 0.623 |
| Age | 0.566 |
| Location | 0.414 |
| Tumor size (cm) | 0.536 |
|  |  |
| Clinical stage | 0.023 |
| Distant Metastasis | 0.009 |

Statistical significance was set at *p* < 0.05

Univariate analyses revealed that sex, age, tumor location, tumor size were not associated with Overall Survival (Supplementary Table1). SNHG16 expression level, clinical stage and distant metastasis was associated with significant differences in Overall Survival (Supplementary Table1).

Supplementary Table2. Multivariate analysis for overall survival of patients with osteosarcoma.

| Variable | Overall Survival | |
| --- | --- | --- |
|  | Hazard ratio (95% CI) | *P* value |
| SNHG16 expression |  |  |
| Low | Reference | 0.044 |
| High | 2.720 (1.028-7.193) |  |
| Clinical stage |  |  |
| I+IIA | Reference | 0.211 |
| IIB/III | 2.294 (0.625-8.417) |  |
| Distant Metastasis |  |  |
| Yes | Reference | 0.030 |
| No | 0.344(0.131-0.904) |  |

Statistical significance was set at *p* < 0.05

On multivariate analysis of all patients, SNHG16 expression level and distant metastasis were determined to be independent risk factors of overall survival (Supplementary Table2).
