# Supplementary figures and images for: LncRNA SNHG16 contributes to osteosarcoma progression by acting as a ceRNA of miR-1285-3p
Source: BMC Cancer. 2021 Apr 6;21:355. doi: 10.1186/s12885-021-07933-2 (PMC8022398; doi:10.1186/s12885-021-07933-2)

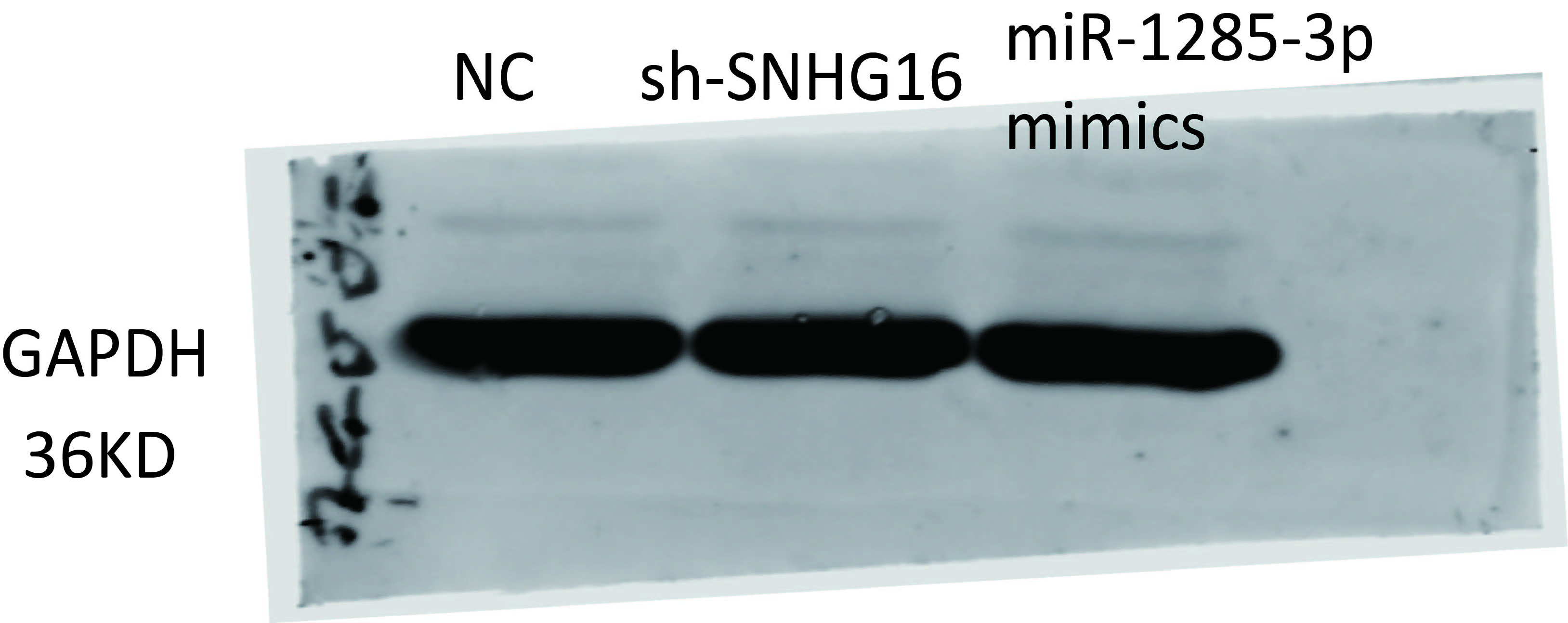

Supplement: Supplementary file 3 — Additional file 3. GAPDH [file 12885_2021_7933_MOESM3_ESM.jpg]

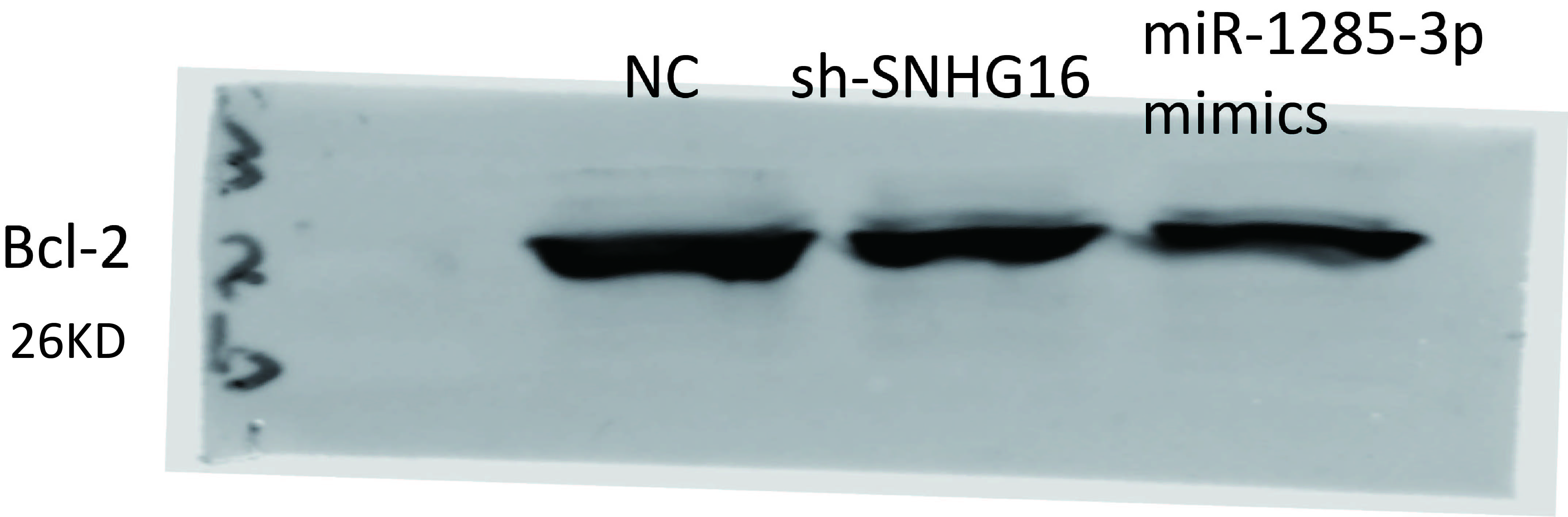

Supplement: Supplementary file 4 — Additional file 4. BCL2 [file 12885_2021_7933_MOESM4_ESM.jpg]

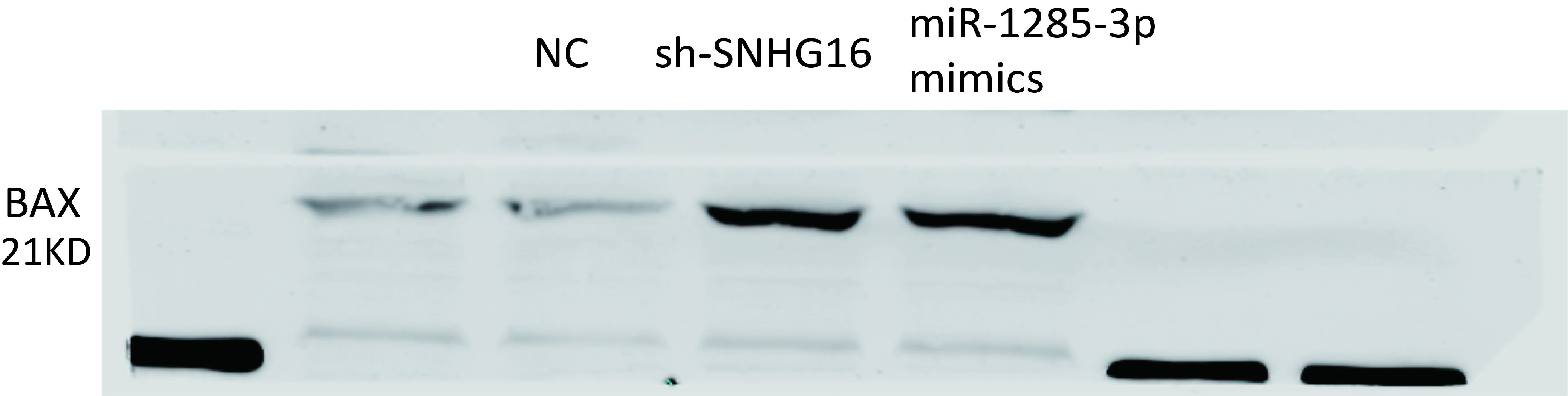

Supplement: Supplementary file 5 — Additional file 5. BAX [file 12885_2021_7933_MOESM5_ESM.jpg]

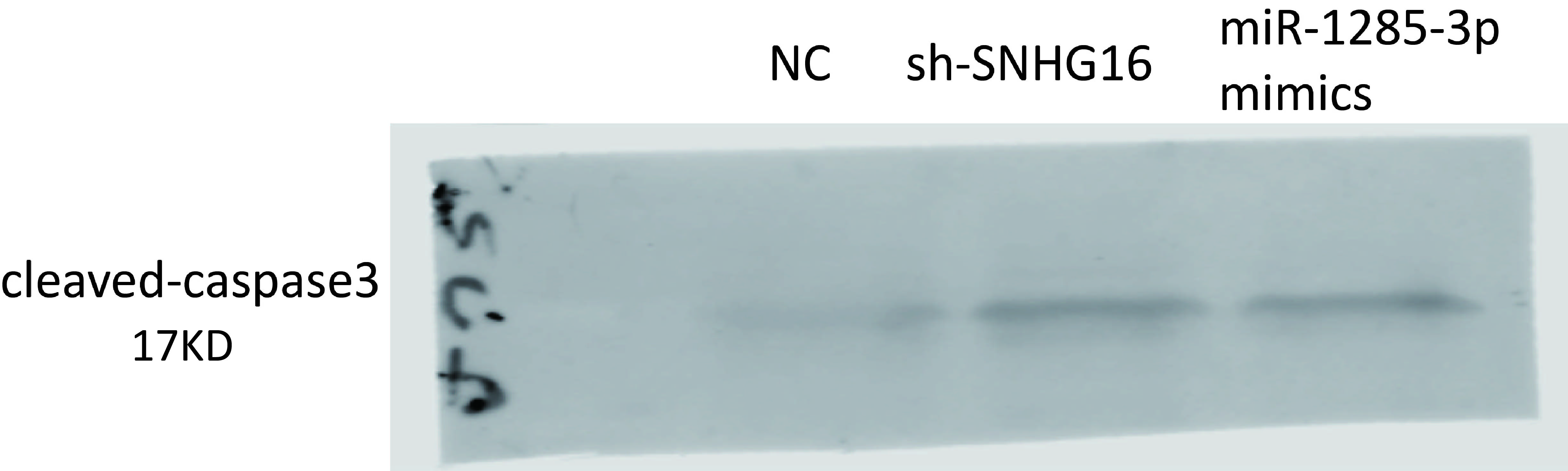

Supplement: Supplementary file 6 — Additional file 6. Pro-caspase [file 12885_2021_7933_MOESM6_ESM.jpg]

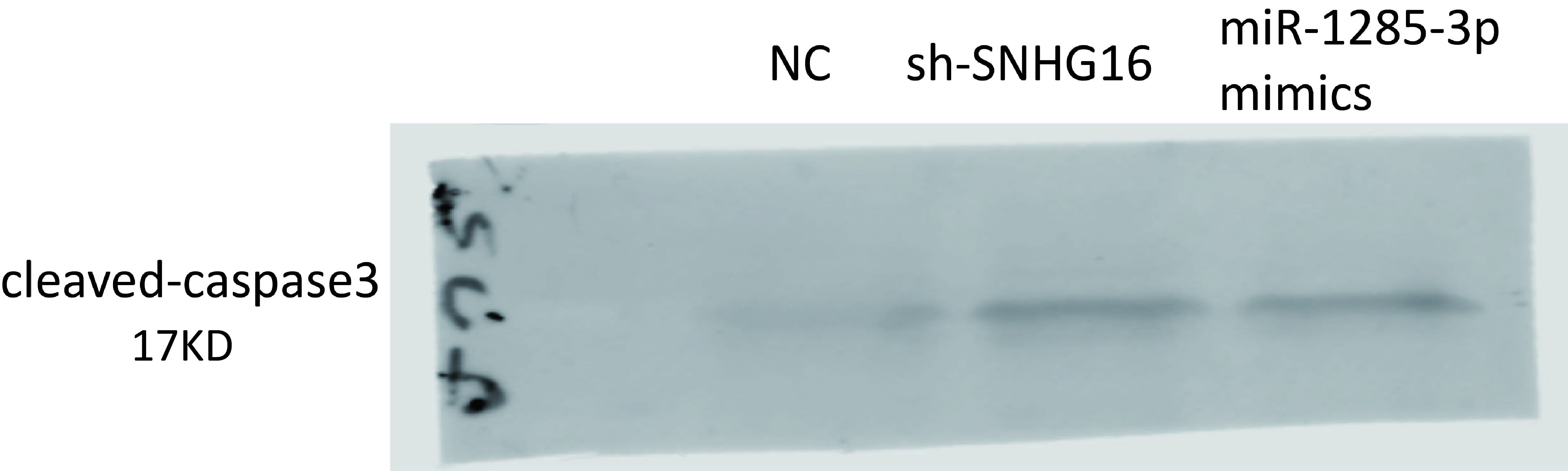

Supplement: Supplementary file 7 — Additional file 7. Cleaved-caspase [file 12885_2021_7933_MOESM7_ESM.jpg]
